# Spatially Correlated Sparse MIMO Channel Path Delay Estimation in Scattering Environments Based on Signal Subspace Tracking

**DOI:** 10.3390/s18051451

**Published:** 2018-05-06

**Authors:** Ali Mohydeen, Pascal Chargé, Yide Wang, Oussama Bazzi, Yuehua Ding

**Affiliations:** 1 Institute of Electronics and Telecommunications of Rennes (IETR), UMR CNRS 6164, Polytech Nantes, Rue Christian Pauc, BP 50609, 44306 Nantes CEDEX 3, France; ali.mohydeen@univ-nantes.fr (A.M.); pascal.charge@univ-nantes.fr (P.C.); yide.wang@univ-nantes.fr (Y.W.); 2 Department of Physics and Electronics, Faculty of Sciences 1, Lebanese University, Hadath, Beirut 14/6573, Lebanon; obazzi@ul.edu.lb; 3 School of Electronic and Information Engineering, South China University of Technology, Wushan Road, Tianhe District, Guangzhou 510641, China

**Keywords:** MIMO, channel delay estimation, subspace tracking

## Abstract

A parametric scheme for spatially correlated sparse multiple-input multiple-output (MIMO) channel path delay estimation in scattering environments is presented in this paper. In MIMO outdoor communication scenarios, channel impulse responses (CIRs) of different transmit–receive antenna pairs are often supposed to be sparse due to a few significant scatterers, and share a common sparse pattern, such that path delays are assumed to be equal for every transmit–receive antenna pair. In some existing works, an exact common support condition is exploited, where the path delays are considered equal for every transmit–receive antenna pair, meanwhile ignoring the influence of scattering. A more realistic channel model is proposed in this paper, where due to scatterers in the environment, the received signals are modeled as clusters of multi-rays around a nominal or mean time delay at different antenna elements, resulting in a non-strictly exact common support phenomenon. A method for estimating the channel mean path delays is then derived based on the subspace approach, and the tracking of the effective dimension of the signal subspace that changes due to the wireless environment. The proposed method shows an improved channel mean path delays estimation performance in comparison with the conventional estimation methods.

## 1. Introduction

Multiple-input multiple-output (MIMO) technology has become an active research topic during the last decade due to its capability for achieving the high transmission rates required by an increasing number of data-demanding applications. MIMO technology provides a plenty of benefits that allow dealing with the challenges posed by the impairments in the wireless channel, especially multipath fading [1]. It provides several important performance gains such as antenna gain, diversity gain, and multiplexing gain. The benefits of MIMO are achieved through the exploitation of the spatial dimension across multiple antennas at the transmitter and receiver, in addition to the time and frequency dimensions already exploited in the conventional single-input single-output (SISO) systems [2]. However, a performant “ideal” MIMO communication system would require an exact knowledge of the MIMO channel or channel state information (CSI). MIMO channel parameters estimation is required for the equalization at the receiver side and precoding at the transmitter side. Hence, channel parameter estimation is a crucial part in MIMO communication. Channel estimation methods are divided into nonparametric and parametric categories. Nonparametric methods are unconstrained methods that exploit no or few assumptions about the channel model, such that they have no associated parameters to rely on, resulting in an increase in the dimensions of the estimation problem. On the other hand, parametric methods are associated with parametric models that are defined by a finite number of parameters with clear physical meanings related to the signal propagation through the channel. Among many other characteristics, some parametric methods exploit the sparsity of wireless channels [3,4,5]. Parametric methods, if wisely used, can achieve robust estimation and decrease the dimensions of the estimation problem.

An accurate MIMO channel model is important to exploit the advantages provided by MIMO systems. This should be done in a realistic way that accurately describes the propagation channel. Recently, the issue of sparsity of the wireless channel has received a lot of attention. It is a fact that, in many scenarios, wireless channels can be characterized by few significant paths due to a few dominant scatterers distributed in the environment, causing a sparse channel impulse response (CIR) [6,7,8,9,10]. This sparsity property has been shown to be reasonable through physical investigations that have been reported for some physical channels [11]. In MIMO outdoor communication scenarios, the CIRs of different transmit–receive antenna pairs share a common support. As the transmit and receive array dimensions are relatively small compared to the long transmission distance, the signals received by the different closely located antennas for each path are spatially correlated [9,12]. In some existing works [9,13], an exact common support condition is considered such that different transmit–receive antenna pairs share exactly the same path delays, meanwhile ignoring the effect of scattering due to the environment. Clustering due to scatterers is an important property that characterizes a wireless channel, such that the channel is modeled as clusters of multi-rays [14,15,16,17]. In this work, in order to match a more realistic channel model, a sparse clustered channel model is considered. The channel is assumed to be sparse in the sense that it contains few significant paths where, due to the physical properties of scatterers, each significant path is modeled as a cluster of multi-rays around a nominal or mean time delay [18,19,20]. This is what we refer to as “delay spreading”. Considering the long distance between the transmit and receive antenna arrays in outdoor scenarios, the above mentioned delay spreading can be assumed to be relatively small. It follows that the multipath clusters can be basically described by their mean delays where it seems neither meaningful nor possible to estimate the time delay of each ray constituting the clusters.

In this paper, a new derivation of the channel model based on higher-order Taylor expansion is proposed. It is worth noting that some previous derivations of the channel model with scattering have been proposed in other contexts (direction of arrival estimation/localization, joint angle and delay estimation) but were limited to the first-order Taylor expansion [18,19,20]. A subspace based method that exploits the derived parametric channel model is then proposed to estimate the mean path delays. The minimum description length (MDL) criterion [21,22] is used to track the signal subspace in order to estimate its effective dimension that is changing due to the wireless environment. Taking into consideration this phenomenon, the proposed method provides an improved channel mean path delays estimation performance in comparison to the conventional subspace based methods which do not take the scattering in the environment into account.

## 2. System and Channel Model

### 2.1. Channel Model

For an N×M MIMO system, considering the models presented in [18,19,20] and focusing on the channel path delays, the CIR $h^{\left(n,m\right)}\left(t\right)$ between the *n*th transmit antenna and *m*th receive antenna can be modeled as:

(1) $$h^{\left(n,m\right)}\left(t\right)=\sum_{l=1}^{L}\sum_{p=1}^{P_{l}}\alpha_{lp}^{\left(n,m\right)}\delta \left(t-(t_{l}+\tau_{lp}^{\left(n,m\right)})\right),\;1\leq n\leq N,1\leq m\leq M$$

where δ(.) is the Dirac function; *L* is the number of propagation paths (clusters), with $P_{l}$ multi-rays for each path (cluster); $\left(t_{l}+\tau_{lp}^{\left(n,m\right)}\right)$ and $\alpha_{lp}^{\left(n,m\right)}$ denote the path delays and path gains of path *l*, respectively, between the *n*th transmit antenna and *m*th receive antenna, where $t_{l}$ is the mean delay associated to path *l* shared by all the transmit–receive antenna pairs, and $\tau_{lp}^{\left(n,m\right)}$ is the delay deviation associated to the *p*th contributing ray of path *l* between the *n*th transmit antenna and *m*th receive antenna.

### 2.2. System Model

In the considered N×M MIMO system, each antenna at the emitter side is transmitting pilot symbols on a different carrier in order to identify the channels associated with different transmit–receive antenna pairs. Consider a known pulse shape g(t) transmitted at the *n*th transmit antenna with a constant rate 1/T through a medium consisting of *L* paths. For the *m*th receive antenna, the received baseband signal is:

(2) $$x^{\left(n,m\right)}\left(t\right)=\sum_{l=1}^{L}\sum_{p=1}^{P_{l}}\alpha_{lp}^{\left(n,m\right)}g\left(t-(t_{l}+\tau_{lp}^{\left(n,m\right)}\right)).$$

For notational convenience, the additive noise is not represented in the above equation.

Applying the discrete Fourier transform (DFT), the Fourier coefficients of the received signal are given by:

(3) $$X^{\left(n,m\right)}\left[k\right]=G\left[k\right]\sum_{l=1}^{L}\sum_{p=1}^{P_{l}}\alpha_{lp}^{\left(n,m\right)}e^{-j\frac{2\pi }{T}k\left(t_{l}+\tau_{lp}^{\left(n,m\right)}\right)}$$

for k=−K/2+1,…,K/2, where *K* is the considered number of Fourier coefficients and G[k] is the DFT of pulse g(t). Note that *K* is an even integer.

### 2.3. Scattering Model with Non-Strictly Exact Common Support

Let $v_{k}\left(t\right)=e^{-j\frac{2\pi }{T}kt}$; Equation (3) can be rewritten as:

(4) $$X^{\left(n,m\right)}\left[k\right]=G\left[k\right]\sum_{l=1}^{L}\sum_{p=1}^{P_{l}}\alpha_{lp}^{\left(n,m\right)}v_{k}\left(t_{l}+\tau_{lp}^{\left(n,m\right)}\right).$$

As the deviations $\tau_{lp}^{\left(n,m\right)}$ are considered small, the $U^{th}$-order Taylor expansion of Equation (4) gives:

(5) $$X^{\left(n,m\right)}\left[k\right]=G\left[k\right]\sum_{l=1}^{L}\sum_{p=1}^{P_{l}}\alpha_{lp}^{\left(n,m\right)}(v_{k}\left(t_{l}\right)+\sum_{u=1}^{U}\frac{{\left(\tau_{lp}^{\left(n,m\right)}\right)}^{u}}{u!}v_{k}^{\left(u\right)}\left(t_{l}\right)+R_{U}\left(\tau_{lp}^{\left(n,m\right)}\right)),$$

where $v_{k}^{\left(u\right)}\left(t_{l}\right)$ is the $u^{th}$-order derivative of $v_{k}\left(t_{l}\right)$. In this last equation, $R_{U}\left(\tau_{lp}^{\left(n,m\right)}\right)$ is the remaining term in the Taylor approximation, which is considered small, such that its impact in the approximation can be neglected.

Equation (5) can be written in the following form:

(6) $$X^{\left(n,m\right)}\left[k\right]=G\left[k\right]\sum_{l=1}^{L}\sum_{u=0}^{U}a_{l,u}^{\left(n,m\right)}v_{k}^{\left(u\right)}\left(t_{l}\right),$$

where

(7) $$a_{l,u}^{\left(n,m\right)}=\sum_{p=1}^{P_{l}}\alpha_{lp}^{\left(n,m\right)}\frac{{\left(\tau_{lp}^{\left(n,m\right)}\right)}^{u}}{u!}.$$

Equation (6) is written in the following matrix form:

(8) $$x^{\left(n,m\right)}=GVa^{\left(n,m\right)},$$

where

(9) $$\begin{array}{c} x^{\left(n,m\right)}={\left[X^{\left(n,m\right)}\left[-K/2+1\right],\ldots ,X^{\left(n,m\right)}\left[K/2\right]\right]}^{T},\\ G=diag\{G[-K/2+1],\ldots ,G[K/2]\},\\ V=[V^{\left(0\right)}V^{\left(1\right)}\ldots V^{\left(U\right)}],\\ V^{\left(u\right)}=\left[v^{\left(u\right)}\left(t_{1}\right),\ldots ,v^{\left(u\right)}\left(t_{L}\right)\right],\\ v^{\left(u\right)}\left(t_{l}\right)={\left[v_{-K/2+1}^{\left(u\right)}\left(t_{l}\right),\ldots ,v_{K/2}^{\left(u\right)}\left(t_{l}\right)\right]}^{T},\\ v_{k}^{\left(u\right)}\left(t_{l}\right)={\left(-j\frac{2\pi }{T}k\right)}^{u}v_{k}\left(t_{l}\right),\\ a^{\left(n,m\right)}={\left[a_{0}^{{\left(n,m\right)}^{T}},\ldots ,a_{U}^{{\left(n,m\right)}^{T}}\right]}^{T},\\ a_{u}^{\left(n,m\right)}={\left[a_{1,u}^{\left(n,m\right)},\ldots ,a_{L,u}^{\left(n,m\right)}\right]}^{T}, \end{array}$$

with $x^{\left(n,m\right)}\in C^{K\times 1}$, $V\in C^{K\times (U+1)L}$, and $a^{\left(n,m\right)}\in C^{(U+1)L\times 1}$.

It is worth noting that matrix V is the same for all the transmit–receive antenna pairs, as the mean delays of each path are assumed to be the same for all the transmit–receive antenna pairs.

The matrix of Fourier coefficient vectors at antenna *m* due to the *N* transmit antennas is given by:

(10) $$X^{\left(m\right)}=\left[x^{\left(1,m\right)}\ldots \;x^{\left(N,m\right)}\right],$$

which can be written as:

(11) $$X^{\left(m\right)}=GVA^{\left(m\right)},$$

where $A^{\left(m\right)}=\left[a^{\left(1,m\right)}\ldots \;a^{\left(N,m\right)}\right]$.

Arranging the Fourier coefficient matrices of different receive antennas as follows:

(12) $$X=[X^{\left(1\right)}X^{\left(2\right)}\ldots \;X^{\left(M\right)}]$$

X can be written as:

(13) $$\begin{array}{c} X=GVA, \end{array}$$

where $A=[A^{\left(1\right)}A^{\left(2\right)}\ldots \;A^{\left(M\right)}]$.

Multiplying Equation (13) by the inverse of G, we have:

(14) Y=VA.

### 2.4. Second Order Statistical Analysis

The covariance matrix $R_{Y}$ of the observation Fourier coefficient vectors Y could be estimated as:

(15) $$R_{Y}=\frac{1}{NM}YY^{H}.$$

For the noise-free case:

(16) $$R_{Y}=VR_{a}V^{H},$$

where the covariance matrix $R_{a}$ is calculated as:

(17) $$R_{a}=\frac{1}{NM}\sum_{n=1}^{N}\sum_{m=1}^{M}a^{\left(n,m\right)}a^{{\left(n,m\right)}^{H}}.$$

Assuming that $t_{l}$ for l=1,…,L are different from one another, with K>(U+1)L and NM>(U+1)L, it can be noted from the definition of matrix V that the dimension of its column space is equal to (U+1)L with a full rank property. It follows that the dimension of the signal subspace is equal to the rank of $R_{a}$. In the following we will show that $R_{a}$ is a full rank matrix of rank equal to (U+1)L, which implies that the dimension of the signal subspace is (U+1)L.

**Proof** **Elements.** Consider one path (L=1) of mean delay $t_{1}$; for the sake of simplification, notations are abbreviated as $t_{1}=t$, $P_{1}=P$, $\alpha_{1,p}^{\left(n,m\right)}=\alpha_{p}$, $\tau_{lp}^{\left(n,m\right)}=\tau_{p}$, and $a^{\left(n,m\right)}$ is a (U+1)-length random column vector which is replaced by a new vector a defined as:

(18) $$a={\left[\sum_{p=1}^{P}\alpha_{p}\;\sum_{p=1}^{P}\alpha_{p}\frac{\tau_{p}^{u}}{u!}\cdots \sum_{p=1}^{P}\alpha_{p}\frac{\tau_{p}^{U}}{U!}\right]}^{T},$$

under the following assumptions:

- $\alpha_{p}$ for p∈{1,…,P} are independent complex random variables such that ∀p,q∈{1,…,P}, $E[\alpha_{p}]=0$; $E\left[\alpha_{p}\alpha_{q}^{*}\right]=\sigma_{p}^{2}$ if p=q, 0 otherwise; and $\sum_{p=1}^{P}E[|\alpha_{p}{|}^{2}]=1$.
- $\tau_{p}$ for p∈{1,…,P} are independent real random variables having the same distribution.
- $\alpha_{p}$ and $\tau_{p}$ are independent.

Defining indices u,v∈{0,1…U}, the ${\left(u+1,v+1\right)}^{th}$ element of matrix $E[aa^{H}]$ is given as:

(19) $${\left[E\left[aa^{H}\right]\right]}_{\left(u+1,v+1\right)}=E[\sum_{p=1}^{P}\alpha_{p}\frac{\tau_{p}^{u}}{u!}\sum_{q=1}^{P}\alpha_{q}\frac{\tau_{q}^{v}}{v!}]=\frac{1}{u!v!}\sum_{p=1}^{P}\sum_{q=1}^{P}E\left[\alpha_{p}\alpha_{q}^{*}\tau_{p}^{u}\tau_{q}^{v}\right].$$

It follows that:

(20) $${\left[E\left[aa^{H}\right]\right]}_{\left(u+1,v+1\right)}=\frac{1}{u!v!}\sum_{p=1}^{P}E[|\alpha_{p}{|}^{2}]E\left[\tau_{p}^{u+v}\right]=\frac{E[\tau_{p}^{u+v}]}{u!v!}.$$

For uniformly distributed $\tau_{p}$ between $\left[-\frac{s}{2},\frac{s}{2}\right]$, we have:

(21) $${\left[E\left[aa^{H}\right]\right]}_{\left(u+1,v+1\right)}=\left\{\begin{array}{cc} {\left(\frac{s}{2}\right)}^{u+v}\frac{1}{u!v!}\frac{1}{u+v+1} & \mathrm{if}\;u+v\;\mathrm{is}\;\mathrm{even}\\ 0 & \mathrm{if}\;u+v\;\mathrm{is}\;\mathrm{odd} \end{array}\right..$$

According to the elements of matrix $E[aa^{H}]$, it follows that its determinant is different from zero, $\left(det\left(E\left[aa^{H}\right]\right)\neq 0\right)$ and as it is a square matrix, then it is a full rank matrix with rank equal to U+1. ☐

## 3. Channel Delay Estimation

The model in Equation (14) suggests the possibility of using subspace-based parameter estimation techniques. In the existing works with an exact common support [9,13], subspace methods such as multiple signal classification (MUSIC) [23] and estimation of signal parameter via rotational invariance techniques (ESPRIT) [24] are applied directly to estimate the delays. Although a non-strictly exact common support model is also taken into account in [9], the conventional ESPRIT and MUSIC methods are used with this model as well. Herein, in accordance with the delay spreading phenomenon, the channel model is reformulated, and a new subspace-based method is proposed to cope with such a model. The principle of subspace-based estimation methods is to find the parameters characterizing V that best match the signal subspace, which can be estimated from the measurements, based on the eigendecomposition of the observation covariance matrix. The problem of estimating the correct dimension of signal or orthogonal subspaces is of great importance in such methods, especially in scattering situations where this problem becomes more complicated.

### 3.1. Asymptotic Analysis of the Signal Subspace Dimension

In the exact common support case with no delay spreading, the noise-free covariance matrix $R_{Y}$ is of rank *L*. However, according to the *U*th-order Taylor-approximated model (6), due to the delay spreading, the rank of $R_{Y}$ is increased to (U+1)L, leading to a larger dimension of the signal subspace. More generally, considering the exact model (Equation (4)) with additive noise, most of the energy of the signal will be concentrated in few eigenvalues of the covariance matrix depending on the standard deviation of the delay spreading, and on the level of noise. Due to these factors, different dimensions of the signal subspace can be obtained. It follows that determining the appropriate value of *U* is of great importance, and it should be chosen carefully depending on these factors in order to estimate the so-called effective dimension of the signal subspace. Consequently, based on the definition of matrix V, an appropriate parametric expression of vectors can be chosen in the channel path delays estimation procedure.

### 3.2. Signal Subspace Tracking

From the generalized model in (6), the dimension of the signal subspace is (U+1)L, where *U* is the order of the Taylor expansion that should be estimated to define the effective dimension of the signal subspace. This can be done through analyzing the eigenvalues of the estimated covariance matrix. The MDL and the Akaike information criterion (AIC) [25] are the two most famous information theoretic criteria for estimating the number of sources impinging on an array of sensors [26]. Such methods test the eigenvalues of the estimated covariance matrix where the number of sources is estimated based on minimizing a derived criterion. In general, the MDL is preferred as it is considered to have better performance than AIC [27]. The MDL provides better performance in the presence of spatially and temporally white noise [28]. The AIC provides better estimation in low signal-to-noise ratio (SNR) and small observation sample conditions, however this estimator is not asymptotically consistent. The MDL provides better estimation in the case of large numbers of observation samples, and it is asymptotically consistent. Therefore, the MDL criterion is chosen in this work to estimate the effective dimension of the signal subspace. In fact, the MDL criterion will provide (U+2)L as the dimension of the signal subspace. Despite the fact that the error term $R_{U}\left(\tau_{lp}^{\left(n,m\right)}\right)$ in (5) is assumed to have no effect in describing the effective signal subspace, it will be detected by the MDL criterion as it is not equal to zero. Hence, during the signal subspace tracking process, the effective dimension of the signal subspace is estimated by subtracting *L* from the value provided by the MDL criterion. In fact, for L>1, each of the *L* paths is assumed to occupy the same degrees of freedom (U+1) in the signal subspace, which should give rise to an overall subspace dimension equal to (U+1)L, and hence (U+2)L would be provided by the MDL criterion. However in practice, in the presence of noise, the MDL algorithm will sometimes provide an integer in the set {(U+1)L+1,(U+1)L+2,…(U+2)L}. The proposed solution for this case is to estimate *U* according to the following rule:

(22) $$U=\left\lfloor \frac{MDLV}{L}-1\right\rfloor ,$$

where MDLV is the value obtained by the MDL criterion and $\left\lfloor .\right\rfloor$ is the floor function.

### 3.3. Mean Path Delays Estimation

The covariance matrix of Y is estimated as in (15). As shown before, the delay spreading gives rise to an increase in the signal subspace dimension to (U+1)L, where the value of *U* changes according to the SNR as well as the standard deviation of the delay spreading. It follows that the eigenvectors associated with the (U+1)L largest eigenvalues define the signal subspace. Hence, the eigendecomposition of the estimated covariance matrix provides the noise subspace matrix $U_{n}$, where $U_{n}$ is composed of the K−(U+1)L eigenvectors associated with the K−(U+1)L smallest eigenvalues of $R_{Y}$.

For the case of no delay spreading, the conventional MUSIC can be applied to estimate the delays where the vector v(t) is projected on the noise subspace. In the case of delay spreading, as observed in Equation (6), vectors $v^{\left(u\right)}\left(t\right)$ for u=0…,U define the signal subspace. A new cost function is then proposed, such that vectors $v^{\left(u\right)}\left(t\right)$ are jointly projected onto the estimated noise subspace of dimension K−(U+1)L. Hence, the following cost function can be defined:

(23) $$P\left(t\right)=\frac{1}{\sum_{u=0}^{U}v^{\left(u\right)}{\left(t\right)}^{H}U_{n}U_{n}^{H}v^{\left(u\right)}\left(t\right)}$$

where

(24) $$\begin{array}{c} v^{\left(u\right)}\left(t\right)={\left[v_{-K/2+1}^{\left(u\right)}\left(t\right),\ldots ,v_{K/2}^{\left(u\right)}\left(t\right)\right]}^{T},\\ v_{k}^{\left(u\right)}\left(t\right)={\left(-j\frac{2\pi }{T}k\right)}^{u}v_{k}\left(t\right). \end{array}$$

The derived cost function can be written in the following form:

(25) $$P\left(t\right)=\frac{1}{v{\left(t\right)}^{H}\left(\sum_{u=0}^{U}D_{u}^{H}U_{n}U_{n}^{H}D_{u}\right)v\left(t\right)}$$

where

(26) $$\begin{array}{c} v\left(t\right)=v^{\left(0\right)}\left(t\right),\\ D_{u}=diag\left\{{\left(-j\frac{2\pi }{T}\left(-K/2+1\right)\right)}^{u},\ldots ,{\left(-j\frac{2\pi }{T}K/2\right)}^{u}\right\}. \end{array}$$

The proposed cost function turns to be the conventional MUSIC when setting U=0, which is the case for very small delay spreading (where it tends to be neglected). The proposed technique can be seen as an extension of MUSIC in the delay spreading case.

The algorithm used for the estimation is summarized as follows:
**Algorithm of the proposed method**Number of delays *L* is assumed to be known. Collect the NM DFT-domain vectors to build matrix Y (14).Estimate the covariance matrix $R_{Y}$ (15).Apply the eigenvalue decomposition on $R_{Y}$.Estimate the effective dimension of the signal subspace using the MDL criterion combined with the proposed rule (22).Construct matrix $U_{n}$.For each value of *t*, calculate the cost function P(t)(25). Then, the mean path delays $t_{l}$ are estimated by the positions of the *L* peaks of P(t).


The proposed cost function shows no major difference from the conventional MUSIC in terms of the computational complexity as they share the same operations such as the FFT, $R_{Y}$ matrix construction, eigenvalue decomposition, and the spectral searching. The proposed cost function requires a slight increase in the computational complexity due to the addition of the terms corresponding to the delay spreading, which is the projection of $v^{\left(u\right)}\left(t\right)$ for u=0…,U on the estimated noise subspace.

## 4. Simulation Results

Simulations are carried out for a 24×24 MIMO system but similar conclusions can be obtained with other MIMO system configurations. The proposed method can be used for either smaller or larger scale of MIMO systems. The DFT-domain data are generated according to Equation (4) with K=64 Fourier coefficients. The delay deviations $\tau_{lp}^{\left(n,m\right)}$ of multi-rays within each path *l* for each (n,m) transmit–receive antenna pair are generated according to a uniform distribution and centered at $t_{l}$. The corresponding gains $\alpha_{lp}^{\left(n,m\right)}$ of the multi-rays are modeled as complex Gaussian random variables generated in a way that the effective power of each path is normalized, such that $\sum_{p=1}^{P_{l}}E[|\alpha_{lp}^{\left(n,m\right)}{|}^{2}]=1$, ∀{l,n,m}. The added noise is modeled as complex white Gaussian noise. The estimation performance is assessed from Q=500 independent simulations. The root mean square error (RMSE) of mean path delay estimation is calculated as $\sqrt{\frac{\sum_{l=1}^{L}\sum_{i=1}^{Q}{\left({\hat{t}}_{l}\left(i\right)-t_{l}\right)}^{2}}{QT^{2}L}}$, where ${\hat{t}}_{l}\left(i\right)$ is the estimated mean delay for the *i*th experiment and $t_{l}$ is the true mean delay. A unique delay group L=1 is firstly considered.

**Figure 1:**
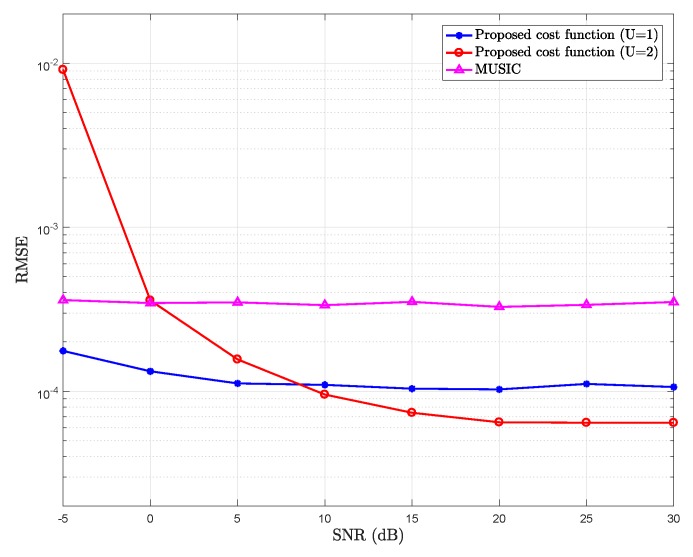
Root mean square error (RMSE) of the mean delay estimation of the proposed cost function for U=1 (K−2L as noise subspace), U=2 (K−3L as noise subspace), and multiple signal classification (MUSIC); $P_{l}=20$, K=64, $\tau_{lp}$ are uniformly distributed over the interval [−0.01,0.01]T (standard deviation of the delay spreading is 0.0058T).

Figure 1 shows the comparison of RMSE of the mean delay estimation versus SNR of the proposed cost function for different values of *U* and MUSIC.

Figure 1 shows that the proposed cost function (assuming U=1) provides a better performance of the mean path delay estimation than the conventional MUSIC.

According to the derivation of the model, in the noise-free case, it is better to choose higher values for *U* to benefit from a better approximation, where small details can be taken into account. On the other hand, with noise-contaminated data, the highest order terms in Taylor expansion (Equation (5)) may be too small with respect to the noise power. Therefore, due to the property of Taylor expansion, it can be noted from Equations (5) and (6), that:

(27) $$a_{l,0}^{\left(n,m\right)}>a_{l,1}^{\left(n,m\right)}>\cdots >a_{l,U}^{\left(n,m\right)}.$$

Hence, for U=2 the elements in vector $a_{l,2}^{\left(n,m\right)}v^{\left(2\right)}\left(t_{l}\right)$ will tend to be quite small. Thus, for a high level of noise, the norm of the vector $a_{l,2}^{\left(n,m\right)}v^{\left(2\right)}\left(t_{l}\right)$ is very small compared to noise. Hence, this part of the signal is dominated by the noise, and it would be better not to consider this part of the signal to represent the signal subspace. For this reason, for low SNR, as can be observed in Figure 1, it would be better to consider U=1 (K−2L as noise subspace). However as SNR increases, it would be worth considering vector $v^{\left(2\right)}\left(\tau_{l}\right)$ as a part of the signal subspace, and then U=2 (K−3L as noise subspace); this can be noted from the figure for SNR ⩾10 dB.

**Figure 2:**
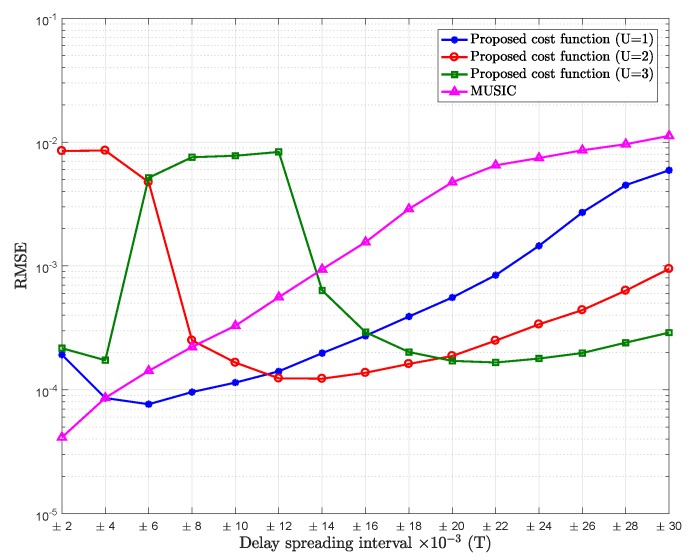
RMSE of mean delay estimation vs. delay spreading of the proposed cost function for U=1 (K−2L as noise subspace), U=2 (K−3L as noise subspace), U=3 (K−4L as noise subspace), and MUSIC; $P_{l}=20$, K=64, signal-to-noise ratio (SNR) = 5 dB.

Figure 2 shows the comparison of RMSE of the mean path delay estimation of the proposed cost function with different values of *U* versus different intervals chosen for the delay spreading, for SNR = 5 dB.

From Figure 2, we can observe that for very small delay spreading with relatively low SNR (SNR = 5 dB), it would be better to consider that the dimension of the signal subspace is one (U=0), for the same reason discussed before, which is the situation of the conventional MUSIC. However, as delay spreading increases, the proposed cost function with U=1 outperforms MUSIC (U=0). It can be also noted that as the delay spreading increases, the proposed cost function for U=3 would tend to outperform the proposed cost function for U=2. The increase in the standard deviation of the delay spreading will give rise to an increase in $a_{l,u}^{\left(n,m\right)}v^{\left(u\right)}\left(t_{l}\right)$. Then, this vector will have greater impact in formulating the signal subspace, hence assuming higher value of *U*.

We can also observe that as the delay spreading increases, the estimation is less accurate, which is probably due to the approximation error of Taylor expansion.

### 4.1. Select U According to the MDL Criterion

**Figure 3:**
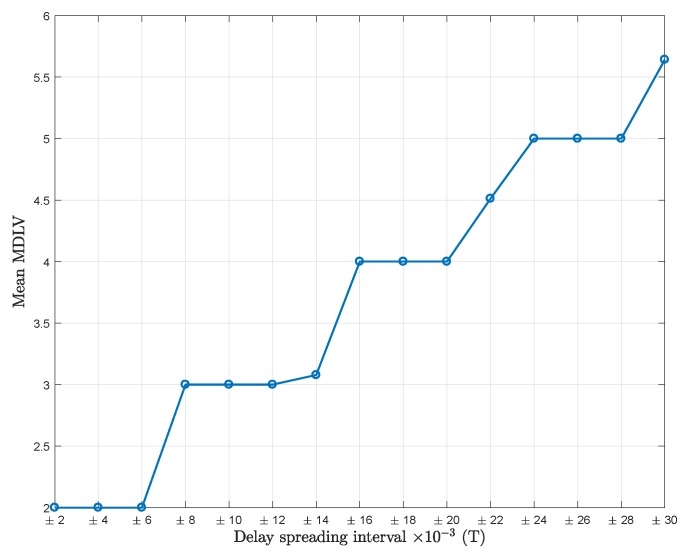
Mean value of the minimum description length (MDL) criterion (mean MDLV) vs. delay spreading; $P_{l}=20$, K=64, SNR = 5 dB, mean MDLV values are obtained from 500 independent simulations each.

To determine the most appropriate value of *U*, the MDL criterion is applied on the eigenvalues of $R_{Y}$. The calculated mean value of the MDL criterion (mean MDLV) versus delay spreading is plotted in Figure 3 to show the performance of the MDL criterion.

Observing Figure 2 and Figure 3 simultaneously, four flat regions can be distinguished. When MDLV = 2, the best choice for the dimension of the signal subspace (U+1)L is 1 (U=0). When MDLV = 3,4,5, the best dimensions of the signal subspace are 2 (U=1), 3 (U=2), and 4 (U=3), respectively.

However, it can be noted that the different flat regions are not always clearly distinguished. For example, when the delay spreading is within [−0.022,+0.022]T, from the observed mean MDLV values, it seems that the MDL criterion is providing four and five values in different realizations. This shows that the MDL criterion, influenced by the random nature of scattering and the relatively high noise level, is not always a foolproof indicator.

**Figure 4:**
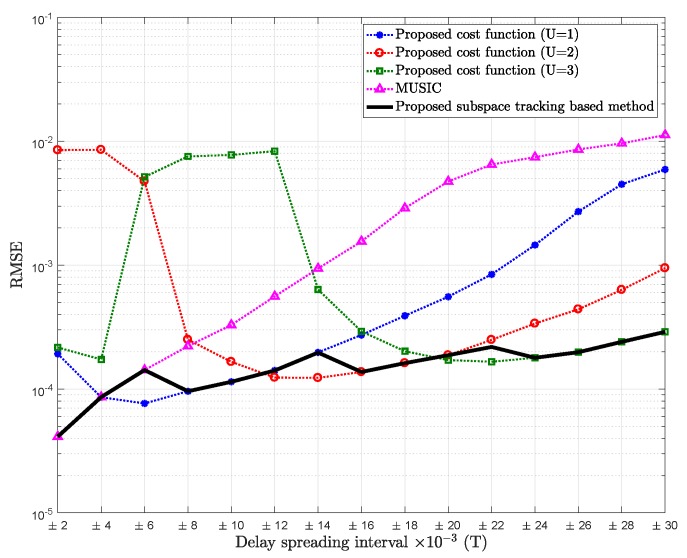
RMSE of mean delay estimation vs. delay spreading of the proposed cost function for U=1, U=2 and U=3, the proposed subspace tracking based method, and MUSIC; $P_{l}=20$, K=64, SNR = 5 dB.

In Figure 4, the RMSE of mean delay estimation of the proposed subspace tracking based method is shown. For each realization of received data, the decision about *U* is obtained from the rule in Equation (22), which is used to estimate the effective dimension of the signal subspace. Then, the proposed cost function (Equation (25)) is applied.

As shown in Figure 4, the proposed method allows for optimal selection of parameter *U* and provides the best mean path delay estimation. However some minor failures of the MDL criterion in systematically estimating the optimal effective dimension of the signal subspace can be noted.

**Figure 5:**
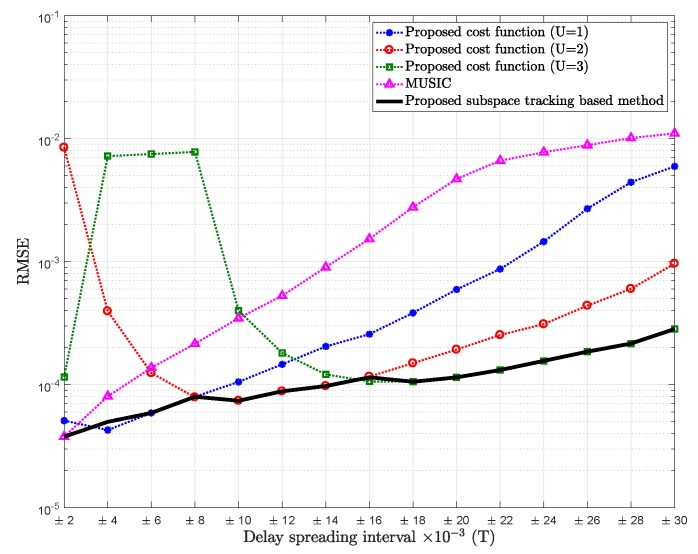
RMSE of mean delay estimation vs. delay spreading of the proposed cost function for U=1, U=2, and U=3, the proposed subspace tracking-based method, and MUSIC; $P_{l}=20$, K=64, SNR = 15 dB.

**Figure 6:**
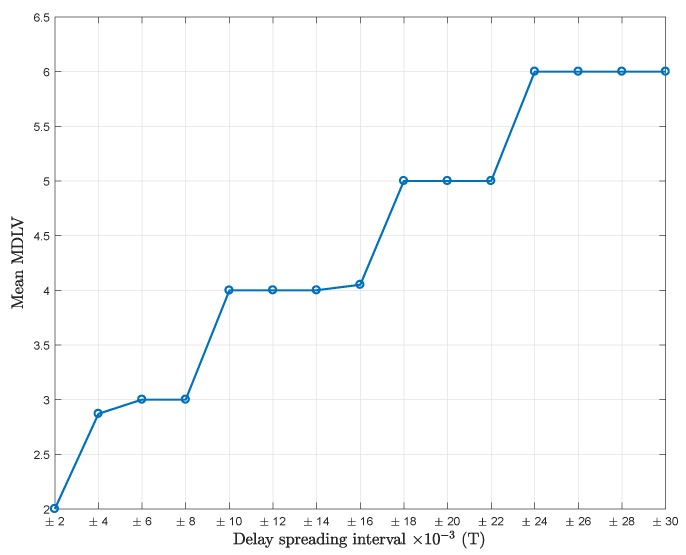
Mean MDLV vs. delay spreading; $P_{l}=20$, K=64, SNR = 15 dB.

Figure 5 and Figure 6 show the RMSE of the mean delay estimation and the mean MDLV versus delay spreading, respectively for SNR =15 dB.

The obtained results show that the MDL criterion provides better estimations of the different effective dimensions of the signal subspace for different delay spreading intervals, and the different regions are well distinguished, leading to an improvement in the mean delay estimation performance of the proposed subspace tracking-based method. As mentioned before, the increase in the RMSE with respect to the delay spreading may be due to the approximation error in Taylor expansion. However, as shown in Figure 4 and Figure 5, this increase is less significant for the proposed subspace tracking-based method; this is the main advantage of tracking the effective dimension of the signal subspace that changes according to the value of the standard deviation of the delay spreading and the noise level.

**Figure 7:**
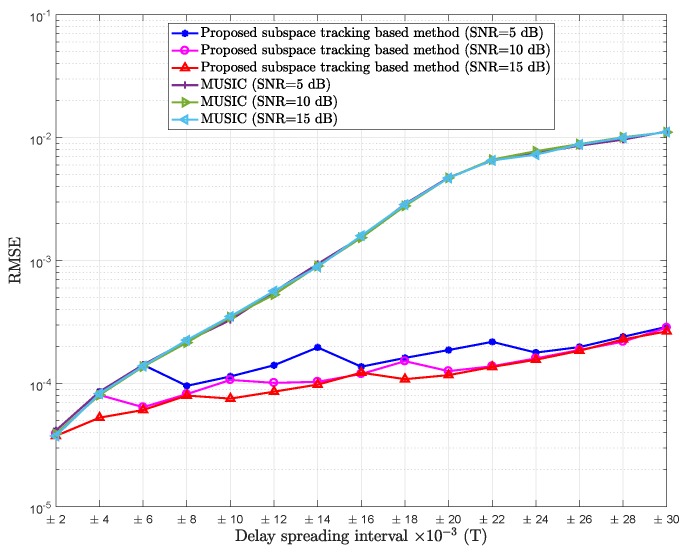
RMSE of mean delay estimation vs. delay spreading of the proposed subspace tracking-based method and MUSIC; $P_{l}=20$, K=64, SNR = 5,10,15 dB.

Figure 7 shows the RMSE of mean delay estimation versus SNR of the proposed subspace tracking based method in comparison with MUSIC for different SNR values.

The obtained results show a moderate improvement in the estimation performance as SNR increases. In fact as SNR increases, better estimation of covariance matrix is obtained, and hence there is better estimation of signal or noise subspaces.

**Figure 8:**
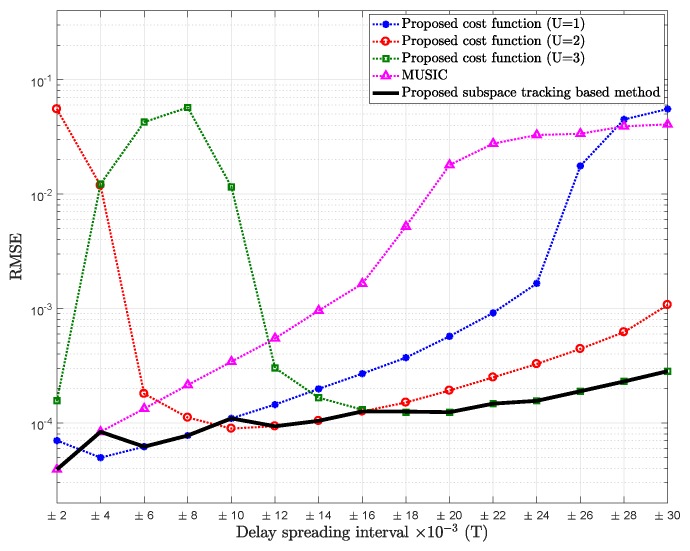
RMSE of mean delay estimation vs. delay spreading of the proposed cost function for U=1, U=2 and U=3, the proposed subspace tracking-based method, and MUSIC; L=2, t=[0.370.51]T, $P_{l}=20$, K=64, SNR = 15 dB.

**Figure 9:**
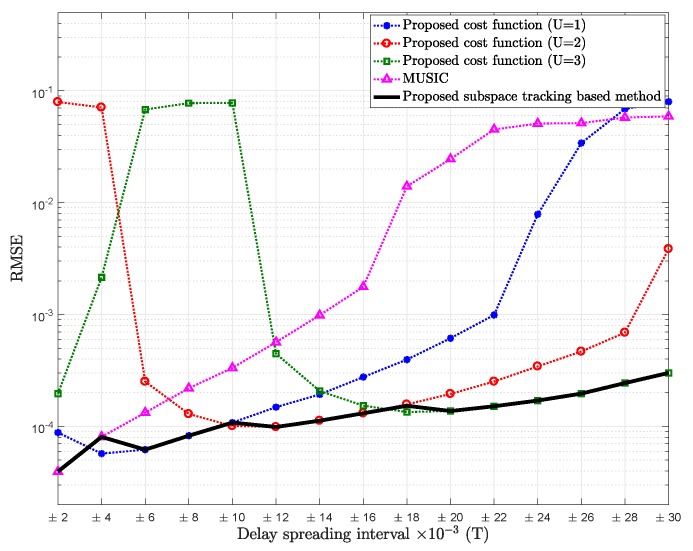
RMSE of mean delay estimation vs. delay spreading of the proposed cost function for U=1, U=2 and U=3, the proposed subspace tracking based method, and MUSIC; L=3, t=[0.370.510.67]T, $P_{l}=20$, K=64, SNR = 15 dB.

As one path delay (L=1) is considered before for the sake of simplification, Figure 8 and Figure 9 show the RMSE of mean path delay estimation for L=2 and L=3 delays, respectively, for SNR = 15 dB.

## 5. Conclusions

A parametric subspace-based estimation method is proposed in this paper to deal with a non-strictly exact common support MIMO channel model that exhibits delay spreading due to scattering. A parametric model of the MIMO channel is first derived. Then, a subspace-based method is developed based on the tracking of the effective dimension of the signal subspace, which depends on the channel features. The proposed subspace tracking-based method is applied to estimate the channel mean delays. The proposed method shows better performance in comparison to the conventional methods, and allows estimating channel mean path delays with more accuracy, leading to a better MIMO channel path delay estimation in scattering environments.

## Abbreviations

The following abbreviations are used in this manuscript:

MIMO	multiple-input multiple-output
CIR	channel impulse response
CSI	channel state information
DFT	discrete Fourier transform
MUSIC	multiple signal classification
ESPRIT	estimation of signal parameter via rotational invariance techniques
MDL	minimum description length
AIC	Akaike information criterion
SNR	signal-to-noise ratio
RMSE	root mean square error

## References

[1] Sklar B. (1997). Rayleigh fading channels in mobile digital communication systems. I. characterization. IEEE Commun. Mag..

[2] Biglieri E., Calderbank R., Constantinides A., Goldsmith A., Paulraj A., Poor H.V. (2007). MIMO Wireless Communications.

[3] Chen N., Zhang J., Zhang P. (2008). Improved channel estimation based on parametric channel approximation modeling for OFDM systems. IEEE Trans. Broadcast..

[4] Yang B., Letaief K.B., Cheng R.S., Cao Z. (2001). Channel estimation for OFDM transmission in multipath fading channels based on parametric channel modeling. IEEE Trans. Commun..

[5] Van Welden D., Moeneclaey M., Steendam H. Parametric versus nonparametric data-aided channel estimation in a multipath fading environment. Proceedings of the Symposium on Communications and Vehicular Technology.

[6] Sayeed A. Sparse multipath wireless channels: Modeling and implications. https://www.ll.mit.edu/asap/asap_06/pdf/Presentations/15_Sayeed_P.pdf.

[7] Bajwa W.U., Sayeed A., Nowak R. Sparse multipath channels: Modeling and estimation. In Proceeding of the IEEE 13th Digital Signal Processing Workshop and 5th IEEE Signal Processing Education.

[8] Bajwa W.U., Haupt J., Sayeed A.M., Nowak R. (2010). Compressed channel sensing: A new approach to estimating sparse multipath channels. Proc. IEEE.

[9] Barbotin Y., Hormati A., Rangan S., Vetterli M. (2012). Estimation of sparse MIMO channels with common support. IEEE Trans. Commun..

[10] Masood M., Afify L.H., Al-Naffouri T.Y. (2015). Efficient coordinated recovery of sparse channels in massive MIMO. IEEE Trans. Signal Proc..

[11] Zhou Y., Herdin M., Sayeed A.M., Bonek E. Experimental Study of MIMO Channel Statistics and Capacity via the Virtual Channel Representation. https://www.researchgate.net/profile/Akbar_Sayeed/publication/244426534_Experimental_Study_of_MIMO_Channel_Statistics_and_Capacity_via_Virtual_Channel_Representation/links/5571b9ee08ae75215866fabb.pdf.

[12] Cho Y.S., Kim J., Yang W.Y., Kang C.G. (2010). MIMO-OFDM Wireless Communications with MATLAB.

[13] Gao Z., Dai L., Lu Z., Yuen C., Wang Z. (2014). Super-resolution sparse MIMO-OFDM channel estimation based on spatial and temporal correlations. IEEE Commun. Lett..

[14] Bonek E. MIMO propagation channel modeling. Proceedings of the 7th European Conference on Antennas and Propagation (EuCAP).

[15] Gustafson C., Haneda K., Wyne S., Tufvesson F. (2014). On mm-wave multipath clustering and channel modeling. IEEE Trans. Antennas Propag..

[16] He R., Chen W., Ai B., Molisch A.F., Wang W., Zhong Z., Yu J., Sangodoyin S. (2016). On the Clustering of Radio Channel Impulse Responses Using Sparsity-Based Methods. IEEE Trans. Antennas Propag..

[17] Xie H., Gao F., Jin S. (2016). An overview of low-rank channel estimation for massive MIMO systems. IEEE Access.

[18] Asztély D., Ottersten B., Swindlehurst A.L. A generalized array manifold model for local scattering in wireless communications. Proceedings of the IEEE International Conference on Acoustics, Speech, and Signal Processing.

[19] Zoubir A., Wang Y., Chargé P., Chandran S. (2005). Localization of Scattered Sources. Advances in Direction-of-Arrival Estimation.

[20] Bazzi A., Slock D.T.M., Meilhac L. On Joint Angle and Delay Estimation in the presence of local scattering. Proceedings of the IEEE International Conference on Communications Workshops (ICC).

[21] Rissanen J. (1978). Modeling by shortest data description. Automatica.

[22] Schwarz G. (1978). Estimating the dimension of a model. Ann. Stat..

[23] Schmidt R. (1986). Multiple emitter location and signal parameter estimation. IEEE Trans. Antennas Propag..

[24] Roy R., Kailath T. (1989). ESPRIT-estimation of signal parameters via rotational invariance techniques. IEEE Trans. Acoust. Speech Signal Proc..

[25] Akaike H. (1974). A new look at the statistical model identification. IEEE Trans. Autom. Control.

[26] Wax M., Kailath T. (1985). Detection of signals by information theoretic criteria. IEEE Trans. Acoust. Speech Signal Proc..

[27] Williams D.B., Douglas B., Madisetti V., Williams D.B. (1999). Detection: Determining the number of sources. Digital Signal Processing Handbook.

[28] Cheng W., Zhang Z., Cao H., He Z., Zhu G. (2014). A comparative study of information-based source number estimation methods and experimental validations on mechanical systems. Sensors.

